# Utilization and control of ecological interactions in polymicrobial infections and community-based microbial cell factories

**DOI:** 10.12688/f1000research.7876.1

**Published:** 2016-03-31

**Authors:** Vinoth Wigneswaran, Cristina Isabel Amador, Lotte Jelsbak, Claus Sternberg, Lars Jelsbak

**Affiliations:** 1 Department of Systems Biology, Technical University of Denmark, Kgs. Lyngby, Denmark; 2 Department of Science and Environment, Roskilde University, Roskilde, Denmark

**Keywords:** microbial cell factories, microbial communities, microbe-microbe interactions

## Abstract

Microbial activities are most often shaped by interactions between co-existing microbes within mixed-species communities. Dissection of the molecular mechanisms of species interactions within communities is a central issue in microbial ecology, and our ability to engineer and control microbial communities depends, to a large extent, on our knowledge of these interactions. This review highlights the recent advances regarding molecular characterization of microbe-microbe interactions that modulate community structure, activity, and stability, and aims to illustrate how these findings have helped us reach an engineering-level understanding of microbial communities in relation to both human health and industrial biotechnology.

## Introduction

Most microbial species are embedded within ecological communities containing many species that interact with one another and their physical environment. Virtually all important microbial activities are shaped by interactions between co-existing microbes within mixed-species communities. These interactions (e.g. in the form of physical, chemical, and genetic signals such as cell-cell contact ^1^, metabolite exchange ^2^, and horizontal gene transfer ^3^) control synergistic, antagonistic, or neutral relationships among the interacting partners and are thus responsible for overall community properties such as species composition and function. In addition, microbial interactions may be dynamic and dependent on environmental context, and microbial communities can have different spatial interactive distributions ranging from metabolic interactions between unassociated planktonic cells in the ocean ^4^ and long-distance electrical signaling within microbial communities ^5, 6^ to local cell-cell interactions occurring within surface-attached biofilms ^7^. Furthermore, a series of recent studies have shown that microbe-microbe and microbe-host interactions can also be mediated by small, air-transmittable molecules ^8– 10^.

Given this complexity among microbial interactive processes, it remains a central challenge to improve our understanding of the molecular mechanisms underlying these interaction processes, their combinatorial effects, and how these interactions ultimately modulate the diversity, behaviors, and activities of the individual species within complex microbial communities.

Dissection of the molecular mechanisms of species interactions within communities is an important issue in microbial ecology. Recently, studies of a diverse range of microbial ecosystems have provided new insight into this area by combining omics methods with classical microbiology cultivation techniques. These systems include multispecies microbial communities formed during the production of fermented food ^11, 12^, microbial communities in acid mine drainages and other polluted habitats ^13^, the commensal microbiota of corals ^14^, as well as several other ecosystems. In this review, we focus primarily on studies of microbe-microbe interactions in host-associated microbial communities and with respect to the engineering of mixed-species microbial cell factories. We use these two examples to broadly illustrate and discuss how knowledge of species interactions is of importance in relation to our ability to control and utilize microbial systems.

## Advances in studies of pathogen-microbiota interactions

In relation to infectious diseases, it is becoming increasingly clear that interactions between bacterial pathogens and other microbial species present at the infection site (for example, co-infecting pathogens or commensal bacteria) can influence disease phenotype or clinical outcome. One example of the importance of such pathogen-microbiota interactions is the well-established role of the intestinal commensal microbiota regarding the prevention of colonization of invading microorganisms including bacterial pathogens in a process known as colonization resistance ^15^. The ability to characterize microbial community structures using 16S ribosomal RNA (rRNA)-based phylogenies or full metagenomic sequencing has now resulted in a much deeper understanding of the interplay between the human microbiome and bacterial pathogens with respect to infectious disease development. For example, studies of the microbial communities in certain chronic infections such as cystic fibrosis (CF) have revealed clear correlations between loss of community diversity and disease progression ^16– 18^. CF patients are predisposed to airway infections from a number of bacterial opportunistic pathogens, among which *Pseudomonas aeruginosa*, *Staphylococcus aureus*, *Haemophilus inﬂuenzae,* and *Burkholderia cepacia* complex (BCC) have been directly associated with CF lung disease ^19– 21^. However, recent studies based on culture-independent methods have demonstrated the presence of many additional bacterial species previously undetected by culture and have revealed a greater microbial diversity in CF airways than previously recognized ^20^. CF airways clearly represent a complex and diverse polymicrobial ecosystem, and, as the disease symptoms become more severe, the CF lung microbiota becomes dominated by the primary pathogen (which most often is the opportunistic pathogen *P. aeruginosa*) ^16– 18^. These results are suggestive of a wider role of the respiratory microbiota and highlight the importance of interactions between the primary pathogen and the microbiota in relation to disease progression.

There are several recent and parallel examples of interactions between the commensal microbiota and possible pathogens which are responsible for limiting colonization and infections by bacterial pathogens such as *Staphylococcus aureus* in the nasal cavity ^22^ and enteropathogenic *Escherichia coli* ^23^ and *Vibrio cholera* ^24^ in the gut. Despite these exciting observations, we are still far from being able to efficiently harness the protective capability of the commensal microbiota against pathogens. Nevertheless, these and related findings clearly point toward chemical and/or biological interference with microbial interaction networks within diseased hosts as alternative treatment strategies against pathogens.

The findings mentioned above highlight the importance of research aimed at systematic mapping of interspecies interactions regarding different types of bacterial infections in combination with the identification and molecular characterization of these interactions. In other words, it is now critical to move beyond correlative research and studies focused on generating microbiome “parts” lists and to instead begin to focus on causality and function at the molecular level. Indeed, a few pioneering studies have recently illustrated these points very clearly, and there are now clear examples of identified microbe-microbe interactions mediated by bacterial metabolites and gene products that function either to limit pathogen colonization ^22– 25^ or to potentiate pathogen expansion or virulence ^26– 28^. Although it is obviously challenging to identify and characterize microbial interspecies interactions in infected hosts, interdisciplinary approaches that combine classical microbiological *in vitro* cultivation techniques with advancing technologies such as three-dimensional (3D) printing ^29^, imaging mass spectrometry ^28, 30^, and development of realistic and controllable *in vitro* model systems ^31^ now make it possible to begin systematically teasing apart the interactions among cultivated key community members and to determine how these interactions modify pathogen behaviors.

## Engineering synthetic multispecies communities for bioproduction purposes

In nature, microbes form interacting mixed-species communities to accomplish complex chemical conversions through division of labor among the individual organisms. We have successfully harnessed the power of such natural microbial communities in food and other industries for decades ^32, 33^, and this has logically led to the emerging concept of community-based cell factories in which synthetic microbial communities are rationally designed and engineered to produce valuable chemicals. Recent studies have indeed demonstrated the potential value of such engineered mixed-species communities as production platforms. In one recent example, a synthetic mixed-species community of *E. coli* and *Saccharomyces cerevisiae* was engineered to produce complex pharmaceutical molecules including precursors of the anti-cancer drug paclitaxel ^34^. By engineering the two organisms to host specific portions of the biosynthetic pathways, it was possible to construct a co-culture system in which an intermediate metabolite was first produced by *E. coli* and then further functionalized by *S. cerevisiae* to give the final product. This study is the first demonstration of the segregation of long and complex biosynthetic pathways into separate organisms each carrying portions of the pathway, which not only enables parallel optimization of the independent pathway modules but also makes it possible to use the best match between particular pathway modules and specific hosts. In another recent study, a fungal-bacterial community was engineered to convert lignocellulosic biomass into biofuels ^35^. Here, the community contained the fungus *Trichoderma reesei,* which can hydrolyze lignocellulosic biomass into soluble saccharides, and the bacterium *E. coli*, which can metabolize these saccharides into isopropanol. In this example, one species provided the carbon source for the second species, which in turn was able to produce the final product on its own.

It is clear from these and other studies that successful engineering of community-based microbial cell factories relies greatly on our molecular understanding of microbe-microbe interactions and how these influence community assembly, stability, and activity.

## Controlling the stability of community-based cell factories

Unlike their natural counterparts, synthetic communities are often unstable. For example, different growth rates among the constituent organisms and secretion of toxic metabolites during growth can influence the stability of the community and will often lead to single-species domination or extinction of the community ^36^. This general instability of synthetic communities limits their translation into real-world applications in industrial biotechnology, and achieving long-term maintenance of synthetic communities is a significant challenge that must be solved.

Although we still have an incomplete understanding of the multiple competitive and cooperative interactions that control microbial community assembly and activity, many different strategies have been successfully employed to increase the stability of synthetic communities. In the first example described above, Zhou *et al.* ^34^ used knowledge of the metabolic capacities of the constituent organisms to construct a specific environment that favored community stability: *E. coli* can use xylose as a carbon source, but when grown on this carbon source, *E. coli* excretes acetate, which is inhibitory to its own growth. On the other hand, *S. cerevisiae* can use acetate as a carbon source but not xylose. The use of a specific carbon source (in this case xylose) thus created a mutualistic interaction between the two organisms, which in turn stabilized the community.

In the other mixed-species community (containing *T. reesei* and *E. coli*) described in the previous section, Minty *et al.* ^35^ took advantage of the particular co-operator/cheater relationship that existed in their engineered fungal-bacterial community and used ecological theory to establish specific conditions (in terms of population sizes) that could stabilize this interaction.

However, community-stabilizing culture conditions—similar to the ones described in these two examples—may be difficult to design and construct for other synthetic communities. Most likely, it is reasonable to expect that alternative approaches will be required in most other situations. These alternative methods may include the construction of synthetic interactions by genetic engineering of the participating species to enforce their interaction. For example, genetic construction of pairs of auxotrophs that cross-feed and support the growth of one another when co-cultured has been shown to be an effective approach for improving community maintenance ^37, 38^. Other strategies have relied on programming specific mutualistic interactions by means of synthetic intercellular signaling circuits ^39– 41^. However, such synthetic interactions are of course also targets of evolutionary process and the long-term stability of these genetic modifications is currently not well understood.

## Form and function in microbial communities

A fundamental principle in biology is that structure (form) and function are inseparable elements. For example, spatial separation of cells that are then subsequently linked together through controlled proximity is an organizational theme frequently observed at all levels in biology ^42^. In relation to natural microbial communities, it is well established that spatial organization of the component species has significant impact on the function and activity of the systems ^43– 45^. Interestingly, such structure/function considerations are often not included in the design of synthetic microbial communities or considered in relation to human infections where the spatial and dynamic distribution of bacteria (including pathogens) and their activities within the human host have been found to be more complex than previously realized ^46– 48^.

Regarding the construction of community-based cell factories, it is certainly a possibility that alternative community-stabilizing methods should build on knowledge of structure/function relationships. Indeed, it has been shown that spatial separation and artificial positioning of cells within synthetic microbial communities improve community function and stability ^36^. Recent advances in fluidics-based bacterial cultivation chambers ^49^, 3D printing methods ^29^, and other micro-patterning techniques ^50^ represent exciting areas in this direction that may advance our ability to efficiently design and control the spatial organization of cells within microbial communities.

## Summary

As either members of infection communities within colonized hosts or part of synthetic communities for sustainable bioproduction, both pathogenic and industrially relevant bacteria are placed in polymicrobial environments in which interactions and spatial position modulate their activity. In both areas, there is a clear need to move beyond the current sequenced-based technologies often used to characterize complex microbial communities and to begin to identify and characterize the function of microbial interactions and the role of spatial organization. The examples shown here illustrate that such knowledge can provide new strategies for better control of bacterial infection and optimized utilization of community-based microbial cell factories. Finally, we emphasize that although our discussion is focused on examples of multispecies bacterial systems in relation to disease and biosynthesis, we believe these are indeed representative examples of an awakening field within microbial ecology focused on understanding species interactions in many types of polymicrobial ecosystems.

## References

[1] Dubey GP Ben-Yehuda S: Intercellular nanotubes mediate bacterial communication. *Cell.* 2011;144(4):590–600. 10.1016/j.cell.2011.01.015 21335240

[2] Møller S Sternberg C Andersen JB: *In situ* gene expression in mixed-culture biofilms: evidence of metabolic interactions between community members. *Appl Environ Microbiol.* 1998;64(2):721–732. 9464414 10.1128/aem.64.2.721-732.1998 PMC106108

[3] Stecher B Denzler R Maier L: Gut inflammation can boost horizontal gene transfer between pathogenic and commensal *Enterobacteriaceae*. *Proc Natl Acad Sci U S A.* 2012;109(4):1269–1274. 10.1073/pnas.1113246109 22232693 PMC3268327

[4] McCarren J Becker JW Repeta DJ: Microbial community transcriptomes reveal microbes and metabolic pathways associated with dissolved organic matter turnover in the sea. *Proc Natl Acad Sci U S A.* 2010;107(38):16420–16427. 10.1073/pnas.1010732107 20807744 PMC2944720

[5] Prindle A Liu J Asally M: Ion channels enable electrical communication in bacterial communities. *Nature.* 2015;527(7576):59–63. 10.1038/nature15709 26503040 PMC4890463

[6] Pfeffer C Larsen S Song J: Filamentous bacteria transport electrons over centimetre distances. *Nature.* 2012;491(7423):218–221. 10.1038/nature11586 23103872

[7] Jelsbak L Søgaard-Andersen L: Pattern formation by a cell surface-associated morphogen in *Myxococcus xanthus*. *Proc Natl Acad Sci U S A.* 2002;99(4):2032–2037. 10.1073/pnas.042535699 11842199 PMC122314

[8] Létoffé S Audrain B Bernier SP: Aerial exposure to the bacterial volatile compound trimethylamine modifies antibiotic resistance of physically separated bacteria by raising culture medium pH. *MBio.* 2014;5(1):e00944–13. 10.1128/mBio.00944-13 24399857 PMC3884056

[9] Niu Q Huang X Zhang L: A Trojan horse mechanism of bacterial pathogenesis against nematodes. *Proc Natl Acad Sci U S A.* 2010;107(38):16631–16636. 10.1073/pnas.1007276107 20733068 PMC2944701

[10] Chen Y Gozzi K Yan F: Acetic Acid Acts as a Volatile Signal To Stimulate Bacterial Biofilm Formation. *MBio.* 2015;6(3):e00392. 10.1128/mBio.00392-15 26060272 PMC4462622

[11] Wolfe BE Button JE Santarelli M: Cheese rind communities provide tractable systems for *in situ* and *in vitro* studies of microbial diversity. *Cell.* 2014;158(2):422–433. 10.1016/j.cell.2014.05.041 25036636 PMC4222527

[12] Sieuwerts S de Bok FA Hugenholtz J: Unraveling microbial interactions in food fermentations: from classical to genomics approaches. *Appl Environ Microbiol.* 2008;74(16):4997–5007. 10.1128/AEM.00113-08 18567682 PMC2519258

[13] Méndez-García C Mesa V Sprenger RR: Microbial stratification in low pH oxic and suboxic macroscopic growths along an acid mine drainage. *ISME J.* 2014;8(6):1259–1274. 10.1038/ismej.2013.242 24430486 PMC4030236

[14] Meyer JL Gunasekera SP Scott RM: Microbiome shifts and the inhibition of quorum sensing by Black Band Disease cyanobacteria. *ISME J.* 2015. 10.1038/ismej.2015.184 26495995 PMC5029210

[15] Bohnhoff M Miller CP: Enhanced susceptibility to *Salmonella* infection in streptomycin-treated mice. *J Infect Dis.* 1962;111(2):117–127. 10.1093/infdis/111.2.117 13968487

[16] Zhao J Schloss PD Kalikin LM: Decade-long bacterial community dynamics in cystic fibrosis airways. *Proc Natl Acad Sci U S A.* 2012;109(15):5809–5814. 10.1073/pnas.1120577109 22451929 PMC3326496

[17] Cox MJ Allgaier M Taylor B: Airway microbiota and pathogen abundance in age-stratified cystic fibrosis patients. *PLoS One.* 2010;5(6):e11044. 10.1371/journal.pone.0011044 20585638 PMC2890402

[18] Blainey PC Milla CE Cornfield DN: Quantitative analysis of the human airway microbial ecology reveals a pervasive signature for cystic fibrosis. *Sci Transl Med.* 2012;4(153):153ra130. 10.1126/scitranslmed.3004458 23019655 PMC3898170

[19] Hoiby N: Epidemiological investigations of the respiratory tract bacteriology in patients with cystic fibrosis. *Acta Pathol Microbiol Scand B Microbiol Immunol.* 1974;82(4):541–550. 10.1111/j.1699-0463.1974.tb02364.x 4153350

[20] Harrison F: Microbial ecology of the cystic fibrosis lung. *Microbiology.* 2007;153(Pt 4):917–923. 10.1099/mic.0.2006/004077-0 17379702

[21] Yang L Jelsbak L Molin S: Microbial ecology and adaptation in cystic fibrosis airways. *Environ Microbiol.* 2011;13(7):1682–1689. 10.1111/j.1462-2920.2011.02459.x 21429065

[22] Iwase T Uehara Y Shinji H: *Staphylococcus epidermidis* Esp inhibits *Staphylococcus aureus* biofilm formation and nasal colonization. *Nature.* 2010;465(7296):346–349. 10.1038/nature09074 20485435

[23] Fukuda S Toh H Hase K: Bifidobacteria can protect from enteropathogenic infection through production of acetate. *Nature.* 2011;469(7331):543–547. 10.1038/nature09646 21270894

[24] Hsiao A Ahmed AM Subramanian S: Members of the human gut microbiota involved in recovery from *Vibrio cholerae* infection. *Nature.* 2014;515(7527):423–426. 10.1038/nature13738 25231861 PMC4353411

[25] Buffie CG Bucci V Stein RR: Precision microbiome reconstitution restores bile acid mediated resistance to *Clostridium difficile*. *Nature.* 2015;517(7533):205–208. 10.1038/nature13828 25337874 PMC4354891

[26] Stacy A Everett J Jorth P: Bacterial fight-and-flight responses enhance virulence in a polymicrobial infection. *Proc Natl Acad Sci U S A.* 2014;111(21):7819–7824. 10.1073/pnas.1400586111 24825893 PMC4040543

[27] Korgaonkar A Trivedi U Rumbaugh KP: Community surveillance enhances *Pseudomonas aeruginosa* virulence during polymicrobial infection. *Proc Natl Acad Sci U S A.* 2013;110(3):1059–1064. 10.1073/pnas.1214550110 23277552 PMC3549110

[28] Frydenlund Michelsen C Hossein Khademi SM Krogh Johansen H: Evolution of metabolic divergence in *Pseudomonas aeruginosa* during long-term infection facilitates a proto-cooperative interspecies interaction. *ISME J.* 2015. 10.1038/ismej.2015.220 26684729 PMC5029194

[29] Connell JL Kim J Shear JB: Real-time monitoring of quorum sensing in 3D-printed bacterial aggregates using scanning electrochemical microscopy. *Proc Natl Acad Sci U S A.* 2014;111(51):18255–18260. 10.1073/pnas.1421211111 25489085 PMC4280622

[30] Moree WJ Phelan VV Wu CH: Interkingdom metabolic transformations captured by microbial imaging mass spectrometry. *Proc Natl Acad Sci U S A.* 2012;109(34):13811–13816. 10.1073/pnas.1206855109 22869730 PMC3427086

[31] Price KE Naimie AA Griffin EF: Tobramycin-Treated *Pseudomonas aeruginosa* PA14 Enhances *Streptococcus constellatus* 7155 Biofilm Formation in a Cystic Fibrosis Model System. *J Bacteriol.* 2015;198(2):237–247. 10.1128/JB.00705-15 26483523 PMC4751783

[32] Smid EJ Lacroix C: Microbe-microbe interactions in mixed culture food fermentations. *Curr Opin Biotechnol.* 2013;24(2):148–154. 10.1016/j.copbio.2012.11.007 23228389

[33] Zhu A Guo J Ni BJ: A novel protocol for model calibration in biological wastewater treatment. *Sci Rep.* 2015;5: 8493. 10.1038/srep08493 25682959 PMC4329560

[34] Zhou K Qiao K Edgar S: Distributing a metabolic pathway among a microbial consortium enhances production of natural products. *Nat Biotechnol.* 2015;33(4):377–383. 10.1038/nbt.3095 25558867 PMC4867547

[35] Minty JJ Singer ME Scholz SA: Design and characterization of synthetic fungal-bacterial consortia for direct production of isobutanol from cellulosic biomass. *Proc Natl Acad Sci U S A.* 2013;110(36):14592–14597. 10.1073/pnas.1218447110 23959872 PMC3767521

[36] Kim HJ Boedicker JQ Choi JW: Defined spatial structure stabilizes a synthetic multispecies bacterial community. *Proc Natl Acad Sci U S A.* 2008;105(47):18188–18193. 10.1073/pnas.0807935105 19011107 PMC2587551

[37] Kerner A Park J Williams A: A programmable *Escherichia coli* consortium via tunable symbiosis. *PLoS One.* 2012;7(3):e34032. 10.1371/journal.pone.0034032 22479509 PMC3316586

[38] Shou W Ram S Vilar JM: Synthetic cooperation in engineered yeast populations. *Proc Natl Acad Sci U S A.* 2007;104(6):1877–1882. 10.1073/pnas.0610575104 17267602 PMC1794266

[39] Brenner K Arnold FH: Self-organization, layered structure, and aggregation enhance persistence of a synthetic biofilm consortium. *PLoS One.* 2011;6(2):e16791. 10.1371/journal.pone.0016791 21347422 PMC3036657

[40] Brenner K Karig DK Weiss R: Engineered bidirectional communication mediates a consensus in a microbial biofilm consortium. *Proc Natl Acad Sci U S A.* 2007;104(44):17300–17304. 10.1073/pnas.0704256104 17959781 PMC2077251

[41] Hong SH Hegde M Kim J: Synthetic quorum-sensing circuit to control consortial biofilm formation and dispersal in a microfluidic device. *Nat Commun.* 2012;3: 613. 10.1038/ncomms1616 22215088 PMC3272573

[42] Gijzen HJ Barugahare M: Contribution of anaerobic protozoa and methanogens to hindgut metabolic activities of the American cockroach, *Periplaneta americana*. *Appl Environ Microbiol.* 1992;58(8):2565–2570. 1514803 10.1128/aem.58.8.2565-2570.1992 PMC195822

[43] Schramm A Larsen LH Revsbech NP: Structure and function of a nitrifying biofilm as determined by *in situ* hybridization and the use of microelectrodes. *Appl Environ Microbiol.* 1996;62(12):4641–4647. 8953735 10.1128/aem.62.12.4641-4647.1996 PMC168290

[44] MacLeod FA Guiot SR Costerton JW: Layered structure of bacterial aggregates produced in an upflow anaerobic sludge bed and filter reactor. *Appl Environ Microbiol.* 1990;56(6):1598–1607. 2383005 10.1128/aem.56.6.1598-1607.1990 PMC184478

[45] Raynaud X Nunan N: Spatial ecology of bacteria at the microscale in soil. *PLoS One.* 2014;9(1):e87217. 10.1371/journal.pone.0087217 24489873 PMC3905020

[46] Markussen T Marvig RL Gómez-Lozano M: Environmental heterogeneity drives within-host diversification and evolution of *Pseudomonas aeruginosa*. *MBio.* 2014;5(5):e01592–14. 10.1128/mBio.01592-14 25227464 PMC4172072

[47] Yan M Pamp SJ Fukuyama J: Nasal microenvironments and interspecific interactions influence nasal microbiota complexity and *S. aureus* carriage. *Cell Host Microbe.* 2013;14(6):631–640. 10.1016/j.chom.2013.11.005 24331461 PMC3902146

[48] Lee SM Donaldson GP Mikulski Z: Bacterial colonization factors control specificity and stability of the gut microbiota. *Nature.* 2013;501(7467):426–429. 10.1038/nature12447 23955152 PMC3893107

[49] Tolker-Nielsen T Sternberg C: Methods for studying biofilm formation: flow cells and confocal laser scanning microscopy. *Methods Mol Biol.* 2014;1149:615–629. 10.1007/978-1-4939-0473-0_47 24818937

[50] Yaguchi T Dwidar M Byun CK: Aqueous two-phase system-derived biofilms for bacterial interaction studies. *Biomacromolecules.* 2012;13(9):2655–2661. 10.1021/bm300500y 22793044

